# Force Eruption of Mandibular Second Incisor in an 11- Year Old Boy: A Technical Report

**Published:** 2013-06

**Authors:** F Sobhnamayan, F Moazami, S Hamedi, R Meshki

**Affiliations:** a Dept. of Endodontics_,_ School of Dentistry, Shiraz University of Medical Sciences, Shiraz, Iran.; b Dept. of Endodontics, School of Dentistry, Bushehr University of Medical Sciences, Bushehr, Iran.; c Dept. of Pedodontics, School of Dentistry, Ahwaz University of Medical Sciences, Ahwaz, Iran.

**Keywords:** Orthodontic Force Eruption, Fractured Teeth, Mandibular Incisor

## Abstract

There is a great challenge in the treatment of deeply fractured and un-restorable teeth among dentists. Orthodontic force eruption is a method of treatment for these teeth to preserve natural root system and periodontal structures. This technical report is a new modification of this procedure presented in an 11- year old boy with deeply fractured left second mandibular incisor. The fractured teeth were treated with root canal therapy and a file #80 was modified to become a hook cemented into the fractured tooth. Anterior teeth were splinted and used as anchorage to help the root extrusion. 1-year follow up of the tooth showed the convenience of the treatment.

This simple and low-cost method can be an acceptable alternative to the current high cost techniques, achieving the same results.

## Introduction

Restoration of deeply fractured teeth often requires interdisciplinary cooperation. Invading the biologic width often leads to the different problems such as chronic gingivitis, bony pockets, loss of clinical attachment and consequently gingival recessions [1-2]. Extraction, surgical crown lengthening, surgical intra-alveolar transplantation and orthodontic force eruption are different methods of treatment in fractured teeth [3]. The best alternative to these surgical approaches are orthodontic force eruption which produce excellent results with good prognosis and low risk of relapse [9]. This technique can be used in deeply fractured teeth even up to 4 mm below the alveolar crest. In order to use this method, the remaining root should be about 12-13mm long [4]. According to these points and in regard to patient's age, orthodontic force eruption was chosen in this particular case.

## Case Report

**Figure 1:**
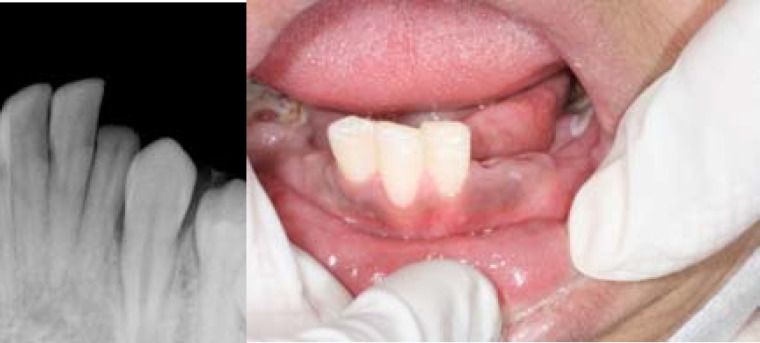
Clinical and radiographic view of fractured incisore

An eleven- year old boy was referred to the Endodontic department of Shiraz Dental School, suffering from a traumatic injury. The injury fractured the tooth #23 about 2mm below the crestal bone (Figure 1). 

After consultation with other dental specialists, orthodontic force eruption was chosen for this case as the overbite and overjet of the boy was normal (overbite=3mm, overjet=2mm).

**Figure 2:**
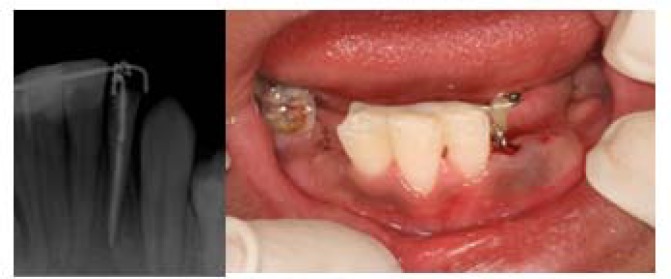
Clinical and radiographic feature of fractured tooth after hook cementation

Endodontic treatment of tooth #23 was performed and 5 mm of gutta-percha in coronal part was removed to provide a space for a hook. The hook was made from a file #80 and cemented in the root canal with poly carboxylate cement (Ariadent; Iran) (Figure 2). Three mandibular anterior teeth were splinted from lingual surfaces with a 0.7- diameter wire (Remanium; Germany) and composite resin (Estelite Quick; Tokuyama Dental Corp, Japan). The wire was rigid enough for force eruption and could tolerate the forces (as we just have one sided anchorage) but not too rigid to cause ankylosis. 

**Figure 3:**
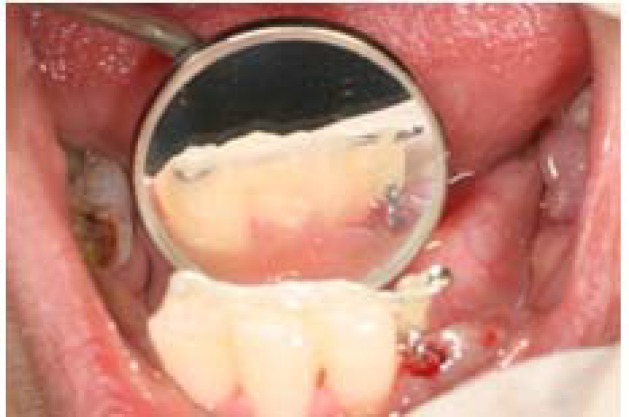
Intra oral feature of splint

The wire was adapted on the lingual surface of the teeth. The distance between the hook and the wire was almost five millimeters to provide enough space for eruption. An elastic loop, size 1/8 (G&H Wire company, USA) was used to connect the hook to the wire (Figure 3). 

**Figure 4:**
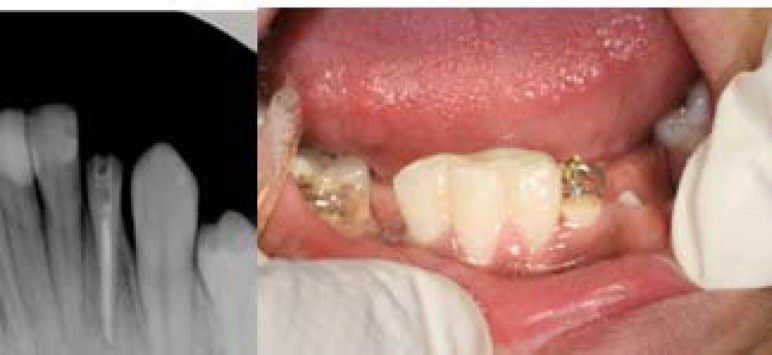
Stabilization period with a rigid wire

The patient was instructed to change the elastic every night and came for follow-up every week. Sulcular incision was done every week. After one month of controlled extrusion, about four millimeters of the root were extruded with an average speed of 1 mm per week. The extruded tooth was retained for two month with a rigid wire with 0.3 mm in diameter (Remanium, Germany) (Figure 4). 

**Figure 5:**
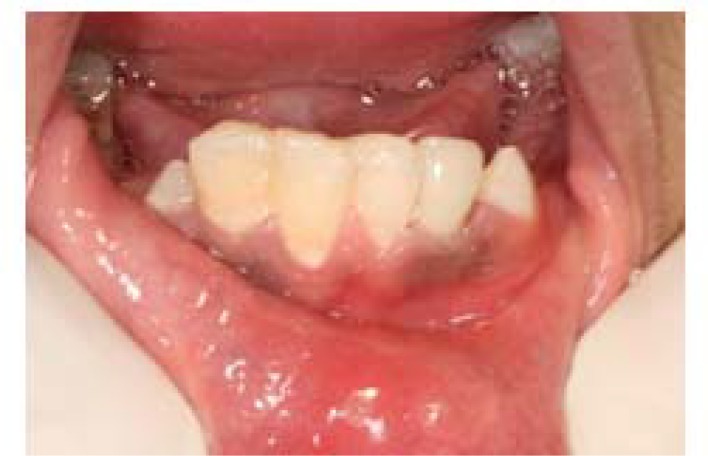
Clinical follow-up after 12 month

After 2 months of stabilization, the tooth was restored. During the 1-year follow-up, the crown was stable and had acceptable function (Figure 5). Radiographic findings showed no signs of resorption or pathological findings in the root region.

## Discussion

Different methods of orthodontic force eruption were introduced and practiced in the different studies [3, 10]. Using orthodontic brackets for extrusion of deeply fractured teeth [11-12] is a common but expensive method which is not applicable for all general dentists. This paper represents a new, simple, effective and also low cost technique, alternative to current methods. 

To avoid the bone and soft tissue movements, rapid extrusion of the tooth was chosen in this case which moved the tooth 3-4 mm per month, unlike the slow extrusion in which the tooth moves only 1-2mm per month.

Rate of extrusion was similar to that recommended by other authors since the force of almost 127 g was applied to extrude the tooth [2-3, 5, 16]. The stabilization period of 7 to 14 weeks is recommended to be adequate in some studies [5-6, 8, 12, and 17]. One month of stabilization for every 1 mm of extrusion is recommended in some studies [18] while others believe in 7 weeks of stabilization [19]. The stabilization period of 8 weeks was chosen in this case which is almost near to other studies and no relapse was shown in the follow-up time.
